# Evaluation of the antioxidant effects of different histone deacetylase inhibitors (HDACis) on human lens epithelial cells (HLECs) after UVB exposure

**DOI:** 10.1186/s12886-019-1056-7

**Published:** 2019-02-04

**Authors:** Xiaodi Qiu, Xianfang Rong, Jin Yang, Yi Lu

**Affiliations:** 1 Eye Institute, Eye and Ear, Nose, and Throat Hospital of Fudan University, 83 Fenyang Road, Shanghai, 200031 People’s Republic of China; 2 Key Laboratory of Myopia, Ministry of Health, Shanghai, 200031 People’s Republic of China; 3 Shanghai Key Laboratory of Visual Impairment and Restoration, Key NHC key Laboratory of Myopia, Fudan University, Shanghai, 200031 People’s Republic of China; 4 Laboratory of Myopia, Chinese Academy of Medical Sciences, Shanghai, 200031 People’s Republic of China

**Keywords:** Histone deacetylase inhibitors, Lens epithelial cells, Ultraviolet-B exposure, Oxidative damage, Beta hydroxybutyrate, Suberoylanilide hydroxamic acid, Trichostatin a, Valproic acid

## Abstract

**Background:**

To compare the protective effects of the histone deacetylase inhibitors (HDACis) β-hydroxybutyrate (βOHB), trichostatin A (TSA), suberoylanilide hydroxamic acid (SAHA) and valproic acid (VPA) on human lens epithelial cells(HLECs) following ultraviolet-B (UVB) exposure.

**Methods:**

HLECs were divided into subgroups: four HDACi groups, a control group, a UVB-treated group and a DMSO group (cells treated with DMSO and UVB irradiation). In the HDACi groups, HLECs were cultured with different concentrations of HDACis 12 h prior to UVB irradiation. The protective effects of the HDACis were evaluated by assessing apoptosis rates, cell activity and expression levels of genes associated with apotosis (caspase-3, Bcl-2, BAX, SOD1, FOXO3A and MT2). The levels of superoxide dismutase (SOD), reactive oxygen species (ROS), malondialdehyde (MDA) and total antioxidant capacity (T-AOC) were detected in order to evaluate oxidative stress.

**Results:**

The results showed that SAHA (1 μmol/L, 2 μmol/L) and TSA (0.2 μmol/L) had mild protective effects on cell viability. βOHB (4 mmol/L) and TSA (0.2 mol/L) demonstrated protective effects on BCL-2 expression. TSA (0.2 mol/L) showed protective effects on SOD1 expression. TSA (0.2 mol/L) and SAHA (1 μmol/L) suppressed BAX and caspase-3 expression. TSA (0.2 mol/L, 0.8 mol/L) and SAHA (1 μmol/L, 2 μmol/L) suppressed the expression of FOXO3A and MT2. SOD levels were increased after treatment with βOHB (4 mmol/L), SAHA (8 μmol/L) and TSA (0.1 mol/L, 0.2 mol/L). T-AOC levels were increased in UVB-treated HLECs after treatment with SAHA (2 μmol/L). MDA levels decreased in UVB-treated HLECs following treatment with TSA (0.2 mol/L, 0.8 mol/L). ROS levels decreased in UVB-treated HLECs following treatment with βOHB (4 mmol/L), SAHA (1 μmol/L, 2 μmol/L) and TSA (0.2 mol/L). Western blotting results demonstrated that SOD1 levels significantly increased in the βOHB (4 mmol/L), SAHA (1 μmol/L, 2 μmol/L), TSA (0.1 mol/L, 0.2 mol/L) and VPA (5 mmol/L) groups. Only SAHA (1 μmol/L) had an anti-apoptotic effect on UVB-treated HLECs.

**Conclusions:**

Our findings indicate that low concentrations of HDACis (1 μmol/L of SAHA) mildly inhibit oxidative stress, thus protecting HLECs from oxidation. These results may suggest that there is a possibility to explore the clinical applications of HDACis for treatment and prevention of cataracts.

## Introduction

Histone (de)acetylation is the most frequent epigenetic modification and has been shown to exert diverse effects on transcriptional activity, interactions between histones and DNA, changes in chromatin structure and the regulation of nucleosomes [1]. The combined action of histone acetyltransferases (HATs) and histone deacetylases (HDACs) mediate the acetylation state of nucleosomal histones [2]. HDACs condense chromatin and reduce gene expression by removing the acetyl group from histones [3]. There had been several HDAC inhibitors (HDACis) which could block the catalytic activity of HDACs [4].

HDACis had been used in psychiatry and neurology as mood stabilizers and anti-epileptics for a long time [5]. Recently, HDACis have drawn attention for potential application in the treatment of neurodegenerative diseases, cancer and inflammatory diseases [6–8]. A previous study from our group found that the decrease of histone acetylation at the SOD1 promoter is associated with the decrease of SOD1 expression in age-related cataracts. Histone acetylation has an important role in regulating SOD1 expression which participate in the pathogenesis of age-related cataracts [9]. In our study, trichostatin A (TSA, an HDAC inhibitor) corrected the anacardic acid (AA, an HAT inhibitor)-induced imbalance between HATs and HDACs, resulting in enhancing SOD1 expression by reversing histone acetylation. The TSA-induced inhibition of proliferation, migration and epithelial mesenchymal transition (EMT) in HLECs maintains lens transparency [10].

Oxidative stress has been shown to play an important role in the pathogenesis of age-related cataracts [11], and disordered antioxidantion has been shown to aggravate cataracts in experimental models [12]. The exposure of Ultraviolet-B (UVB) also contributes to the development of age-related cataracts (ARCs). UVB exposure can alter gene expression, potentially involving epigenetic changes such as histone acetylation and/or DNA methylation [13]. Accordingly, anti-inflammation and antioxidation have been introduced to treat cataracts [11].

Some clinical investigations have indicated that HDACis are generally well tolerated and have antioxidant effects [5, 8, 14]. The ketone body β-hydroxybutyrate (βOHB) has recently been recognized as an specific endogenous inhibitor of class I HDACs [15]. Studies have shown that βOHB treatment increases histone acetylation of FOXO3A and MT2 promoters, and FOXO3A and MT2 genes are also activated by consumption of HDAC1 and HDAC2. Furthermore, treatment of mice with βOHB confers massive protection against oxidative stress [16].

In our study, we explored the protective effects of HDACis on UVB-treated HLECs. We also investigated the intervention related oxidative stress changes in HLECs.

## Materials and methods

### Group setting

Human lens epithelium B3 (HLE-B3) cells were cultured in vitro and divided into seven subgroups for each HDACi as follows. Group 1: normal control group (without UVB irradiation). Group 2: UVB exposure group. Group 3: dimethyl sulfoxide (DMSO) group (cells treated with DMSO, with the same UVB dosage as the UVB group). Groups 4, 5, 6 and 7: different concentrations of HDACis (concentrations 1, 2, 3 and 4). The concentrations of different HDACis were as follows: βOHB (4, 8, 16 and 32 mmol/L), suberoylanilide hydroxamic acid (SAHA) (1, 2, 4 and 8 μmol/L), TSA (0.1, 0.2, 0.4 and 0.8 μmol/L), and valproic acid (VPA) (5, 10, 20 and 40 mmol/L).

### Reagents and antibodies

βOHB, TSA, VPA and SAHA were obtained from Sigma (St Louis, Missouri, USA), dissolved in DMSO, stored at − 20 °C, and thawed before use. Propidium iodide (PI), Annexin V-FITC and DMSO were purchased from Sigma (St Louis). Fetal bovine serum (FBS) and Dulbecco’s modified Eagle’s medium (DMEM)were acquired from Gibco (Invitrogen, Grand Island, NY, USA). Antibodies against Bcl-2, BAX, caspase-3, SOD1, FOXO3A and Metallothionein (MT2) were purchased from Cell Signaling Technology and Santa Cruz Biotechnology (Santa Cruz, California, USA).

### Cell culture, treatment with HDACis and UVB radiation

HLE-B3 cells were purchased from American Type Culture Collection (ATCC; Rockville, MD, CRL-11421) and cultured in DMEM containing 10% FBS in a humidified 5% CO_2_ atmosphere at 37 °C. HLE-B3 cells were cultured in six well plates in FBS-free DMEM for 24 h prior to treatment. Cells were cultured to 70% confluence and subsequently treated with different concentrations of HDACis. Then, HLE-B3 cells were treated with UVB light for 20 min; the UVB dosage was 2 W/m^2^ for 60 min. Control HLECs were treated with DMSO the same concentration of. In preliminary experiments, this concentration of DMSO had no detrimental effect on HLECs. Following treatment, cells were harvested for cell viability assays and for mRNA and protein extraction.

### Cell viability (CCK-8)

Different concentrations of HDACis were first incubated with human lens epithelial cells (HLECs) for 12 h. Cell viability was envaluated with CCK-8 assay according to the manufacturer’s protocol (Dojindo Laboratory, Kumamoto, Japan). Equal amount of HLECs were seeded into 96-well plates in a final volume of 100 μl DMEM per well. After treatment, the samples and 10 μl of CCK-8 kit reagent were co-incubated for 2 h at 37 °C. Absorbance was measured at 450 nm on a multi-well plate reader (Benchmark plus, Bio-Rad, Tokyo, Japan). The percentage of viable cells equation: ratio (%) = [OD (treatment) − OD (the culture medium without cells)]/[OD (control) − OD (the culture medium without cells)] × 100. Each sample was evaluated in six replicates per assay (assays were performed three times).

### Apoptosis assessment

The influence of HDACis on cell apoptosis was quantified using the Annexin-V Fluorescein Isothiocyanate (FITC) Apoptosis Detection Kit I (BD Biosciences, San Diego, CA). Following a 12 h incubation with HDACis and a subsequent UVB exposure, HLECs were dispersed into a single cell suspension using 0.25% trypsin-0.02% EDTA and then washed and labeled with propidium Iodide(PI) and Annexin V-FITC. Cell apoptosis was measured using a flow cytometer (BD Biosciences, San Jose, California, USA). All experiments were performed three times.

### Western blot assay

HLECs were treated with different doses of HDACis and UVB and lysates were prepared from 1 × 10^7^ cells. HLECs were lysed in RIPA buffer (Solarbio, USA), which contained a protease inhibitor cocktail (Beyotime, China) and phenylmethylsulfonyl fluoride (PMSF, Beyotime, China). The supernatant was collected after centrifugation at 12000 rpm for 20 min at 4 °C. The protein concentrations of each sample were measured using a BCA Protein Quantitation Kit (Beyotime, China). All samples were separated in equal amounts by 10% SDS-polyacrylamide gel electrophoresis (PAGE) (100 V for 90 min) and transferred to polyvinylidine difluoride (PVDF) membranes (Millipore, Bedford, MA) using a transfer apparatus (BioRad) at 40 mA for 8 h. Blocking buffer (5% nonfat milk, 200 mM NaCl, 50 mM Tris, 0.05% Tween 20) was used for inhibiting nonspecific protein binding to the membrane. The blocked membranes were then incubated with primary antibodies against GAPDH (1:2000, Abcam), Bax (1:200, Santa Cruz), Bcl2 (1:200, Santa Cruz, USA), caspase-3 (1:500, Abcam, Inc., Cambridge, MA, USA), SOD1 (1:10000, Abcam), FOXO3A (1:5000, Abcam) and MT2 (1:1000, Abcam) at 4 °C overnight. Next, the membrane was washed with TBST (20 mM Tris, 500 mM NaCl, 0.1% Tween 20) at 28 °C for three times (5 min each time) and then incubated with HRP-conjugated secondary antibodies (1:4000; Santa Cruz, USA) at 28 °C for 2 h. Then, the membrane was washed with TBST four times (15 min each time) at 28 °C. The bands were visualized by enhanced chemiluminescence (ECL) (Pierce, Rockford, IL). The film was scanned and measured using ImageQuant software (Molecular Dynamics, Sunnyvale, CA).

### Enzyme-linked immunosorbent assay (ELISA)

HLECs from HDACi-treated groups and controls were used to quantitatively detect SOD1 using a commercially available ELISA (AB Frontier). Absorbance was assessed at 450 nm using the 680XR Microplate reader (Biorad, Hercules, USA). For the SOD1 ELISA, primary antibody was a rabbit monoclonal anti-SOD1 (Sigma) antibody and the secondary antibody was a goat anti-rabbit antibody. The analysis of SOD1 levels was performed by the kit manufacturer. The SOD1 standard curve was generated by linear regression analysis in each group.

### Measurement of oxidative stress

Total antioxidant capacity (T-AOC), reactive oxygen species (ROS), malondialdehyde (MDA) and superoxide dismutase (SOD) levels were quantified according to instructions provided with the detection kits (Jiancheng Bioengineering Institute, Nanjing, China) in order to determine intracellular antioxidant conditions.

### Assessment of mitochondrial membrane potential (MMP)

We prepared JC-1 fluorescent probes (Beyotime, Hangzhou, China) in order to analyze changes in MMP after treatments. The JC-1 solution were incubated with HLECs for 20 min at 37 °C in the dark, and then fluorescence microscopy and flow cytometry were used to analyze MMP., MMP is high under normal conditions and displays red fluorescence. Meanwhile, lower MMP, which is an indicator of early apoptosis, is indicated by green fluorescence. MMP was detected using CellQuest software version 3.3 (BD Biosciences, Franklin Lakes, NJ, USA) and the red:green ratio was calculated.

### Real-time PCR

Trizol Reagent (Invitrogen, Gaithersburg, MD, USA) was used for total RNA isolation and the SuperScript II Reverse Transcriptase kit (Invitrogen, Carlsbad, CA, USA) was used for reverse-transcription. Real-time PCR analysis was performed using the SYBR-Green PCR Master Mix (Applied Biosystems, Foster City, CA, USA). Bcl-2, BAX, caspase-3, SOD1, FOXO3A and MT2 expression levels were shown relative to the indicated control samples after normalized to GADPH levels. All primer sequences are listed in Table 1.

**Table 1:** The primers used for real-time PCR

Gene	Primer F	Primer R	Size (bps)
BCL2	5’ GACTTCGCCGAGATGTCCAG 3’	5’ GCCGGTTCAGGTACTCAGTC 3’	220
BAX	5’ AGACCGTGACCATCTTTGTG 3’	5’ TCCCGAAGGAGGTTTATTACCC 3’	226
caspase 3	5’ AACTGGACTGTGGCATTGAG 3’	5’ ACAAAGCGACTGGATGAACC 3’	161
SOD1	5’ GGTGCTGGTTTGCGTCGTAG 3’	5’ GCCTTCGTCGCCATAACTCG 3’	102
FOXO3a	5’ CCAGGGTAAAGTCAAGTG 3’	5’ GCAGGGTCTCAACATAAG 3’	210
MT2	5’ CCTGCAAATGCAAAGAGTG 3’	5’ ATCCAGGTTTGTGGAAGTC 3’	189
GAPDH	5’ CACCCACTCCTCCACCTTTG 3’	5’ CCACCACCCTGTTGCTGTAG 3’	110

### Data analysis

All statistical analyses were performed using SPSS for Windows (13.0, SPSS, Inc., Chicago, IL, USA). Each value is the mean of at least three separate experiments in each group. The means, standard deviations, and coefficients of variation (CVs) were calculated. Mean values are reported along with the standard deviation of the mean (±SD) unless otherwise stated. Two-sided pairwise comparisons were conducted. Comparisons among experiments were performed by one-way analysis of variance (ANOVA). *P* values < 0.05 were considered statistically significant and those < 0.01 were considered highly significant.

## Results

### HLEC viability and apoptosis following HDACi treatment

HLECs were treated with indicated concentrations of HDACis for 12 h prior to UVB exposure, and then the influence of HDACis on both cell viability and apoptosis were assessed.

**Fig. 1:**
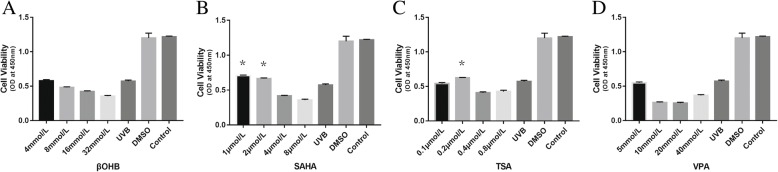
Cell viability of HLECs after HDACi treatment. **a**: βOHB, **b**: SAHA, **c**: TSA, **d**: VPA. βOHB and VPA had no protective effects in UVB-treated HLECs. However, SAHA (1 μmol/L, 2 μmol/L) and TSA (0.2 μmol/L) showed mild protective effects on cell viability after UVB exposure. * *P* < 0.05

CCK-8 assays were used to determine the cell viability of a wide range of HDACi concentrations in HLECs. All the groups of indicated HDACi concentrations were exposed to UVB before CCK-8 assay. βOHB and SAHA showed a dose-dependent decrease in cell viability. βOHB and VPA had no protective effects in UVB-treated HLECs. However, SAHA (1 μmol/L: *P* = 0.007, 2 μmol/L: *P* = 0.023) and TSA (0.2 μmol/L: *P* = 0.031) showed mild protective effects on cell viability after UVB exposure (Fig. 1).

**Fig. 2:**
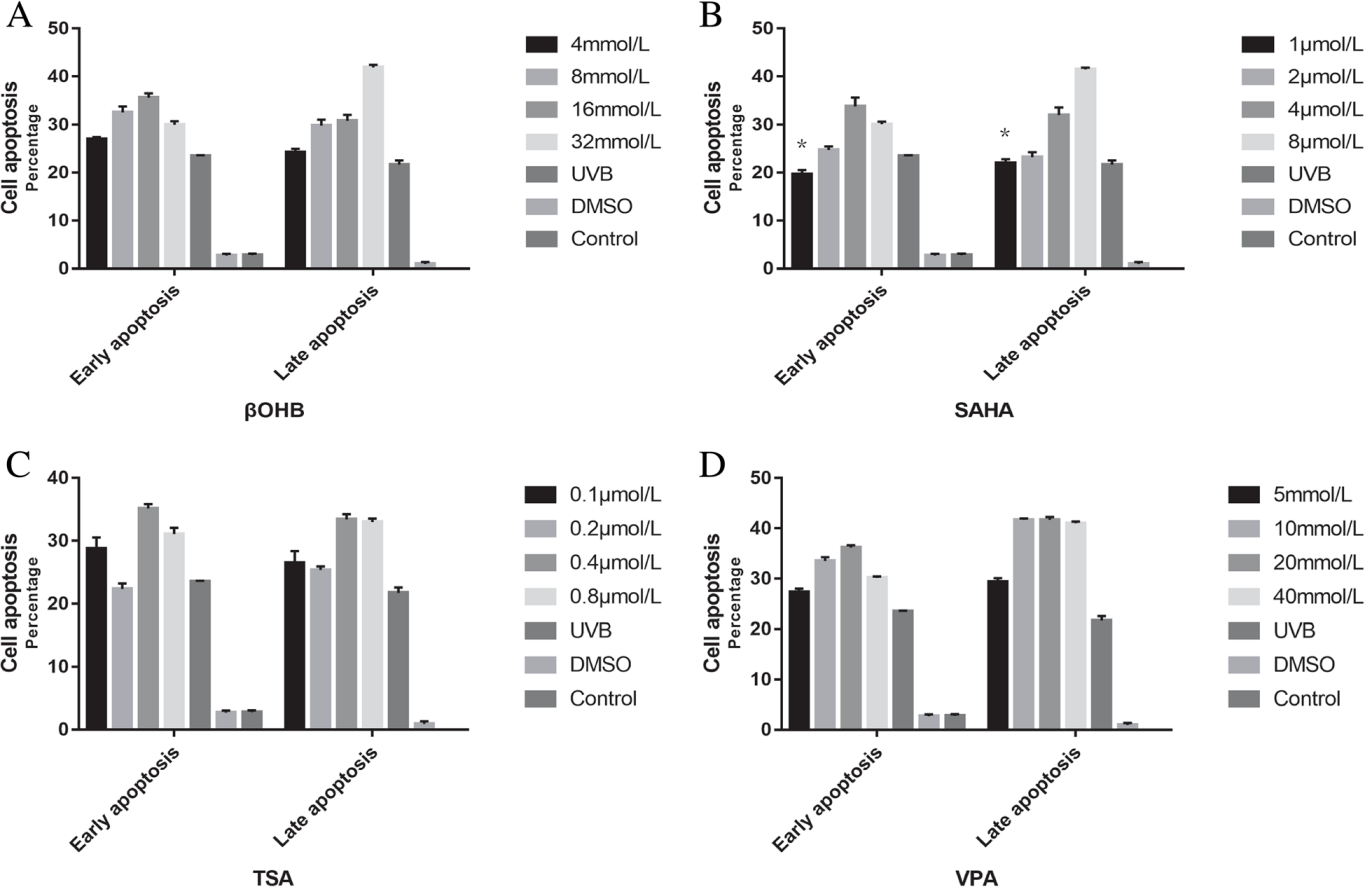
HDACi showed mild anti-apoptosis effect on HLECs after UVB exposure. **a**: βOHB, **b**: SAHA, **c**: TSA, **d**: VPA. The proportion of apoptotic cells increased after UVB exposure. However, only SAHA (1 μmol/L) was able to decrease apoptosis rates in UVB-treated HLECs. Higher concentrations of HDACis resulted in increased levels of cell apoptosis. * *P* < 0.05

We next assessed Apoptosis of HLECs were assessed using Annexin V-FITC/PI flow cytometry. As expected, the proportion of apoptotic cells increased after UVB exposure. However, only SAHA (1 μmol/L: *P* = 0.001) was able to decrease apoptosis rates in UVB-treated HLECs (Fig. 2). Higher concentrations of HDACis resulted in increased levels of cell apoptosis.

### Effects of HDACis on Bcl-2, BAX, caspase-3, SOD1, FOXO3A and MT2 mRNA expression in UVB-treated HLECs

**Fig. 3:**
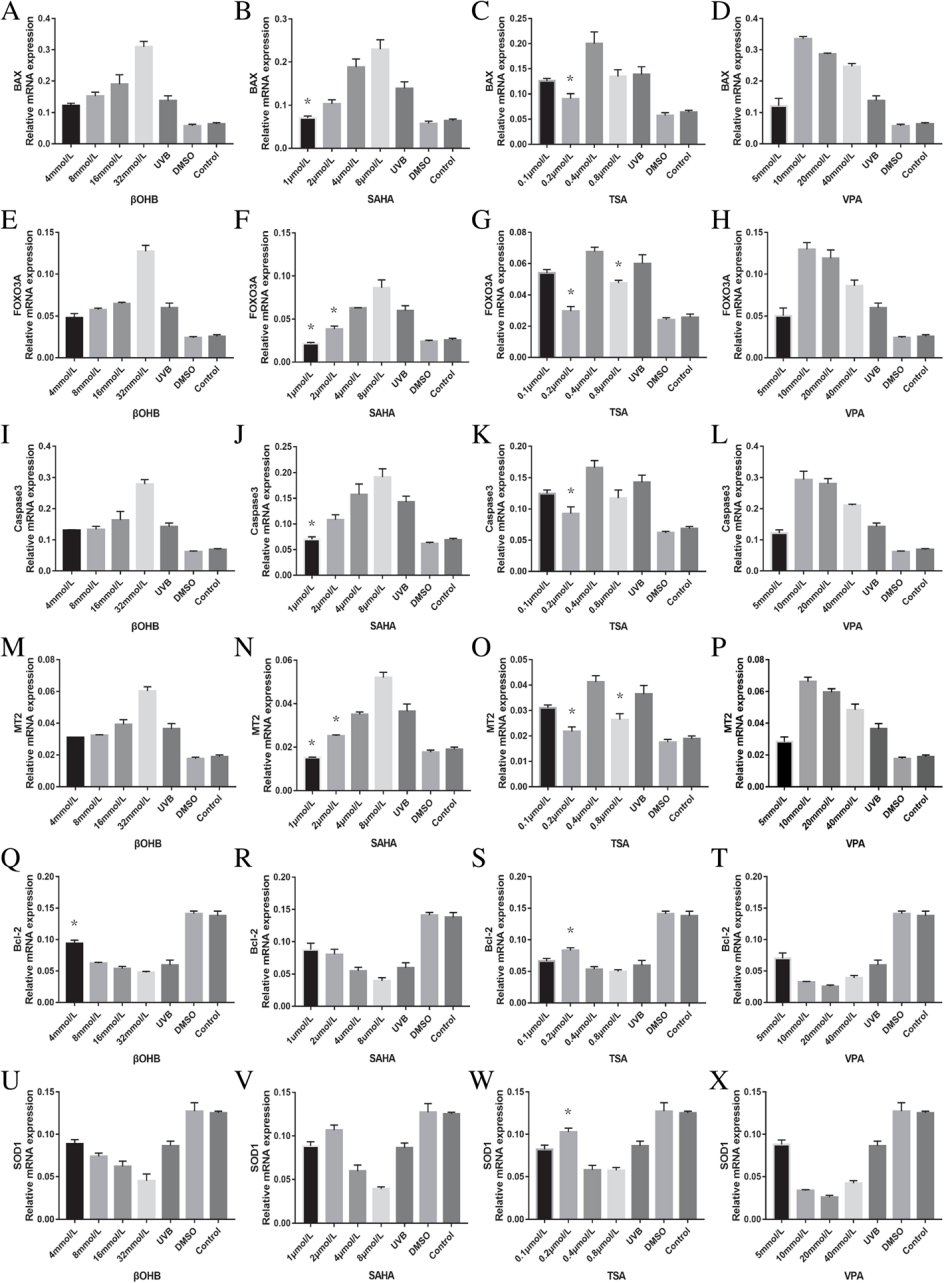
Effects of HDACi on the expressions of Bcl-2, BAX, caspase-3, SOD1, FOXO3A and MT2 mRNA in UVB-treated HLECs. BAX: **a**-**d**, FOXO3A: **e**-**h**, Caspase3: **i**-**l**, MT2:**m**-**p**, Bcl-2: **q**-**t**, SOD1: **u**-**x**. βOHB (4 mmol/L) and TSA (0.2 mol/L) had protective effects on BCL-2 expression. TSA (0.2 mol/L) had a protective effect on SOD1 expression. TSA (0.2 mol/L) and SAHA (1 μmol/L) suppressed BAX and caspase-3 expression. TSA (0.2 mol/L, 0.8 mol/L) and SAHA (1 μmol/L, 2 μmol/L) suppressed the expression of FOXO3A and MT2. The HDACi-induced protective effects were not strictly dose-dependent. * *P* < 0.05

Bcl-2 and SOD1 mRNA levels were appatently suppressed in UVB-treated HLECs (Fig. 3). However, caspase-3, FOXO3A, BAX and MT2 levels were significantly elevated after UVB exposure. βOHB (4 mmol/L: *P* = 0.047) and TSA (0.2 mol/L: *P* = 0.018) had increased the BCL-2 expression. TSA (0.2 mol/L: *P* = 0.024) had increased the on SOD1 expression. TSA (0.2 mol/L: *P*_*BAX*_ = 0.004, *P*_caspase-3_ = 0.000) and SAHA (1 μmol/L: *P*_*BAX*_ = 0.014, *P*_caspase-3_ = 0.005) suppressed BAX and caspase-3 expression. TSA (0.2 mol/L: *P*_FOXO3A_ = 0.003, *P*_MT2_ = 0.024, 0.8 mol/L: *P*_FOXO3A_ = 0.037, *P*_MT2_ = 0.005) and SAHA (1 μmol/L: *P*_FOXO3A_ = 0.010, *P*_MT2_ = 0.009, 2 μmol/L: *P*_FOXO3A_ = 0.021, *P*_MT2_ = 0.026) suppressed the expression of FOXO3A and MT2. The HDACi-induced protective effects were not strictly dose-dependent.

### HDACi attenuates oxidative stress in HLECs after UVB exposure

**Fig. 4:**
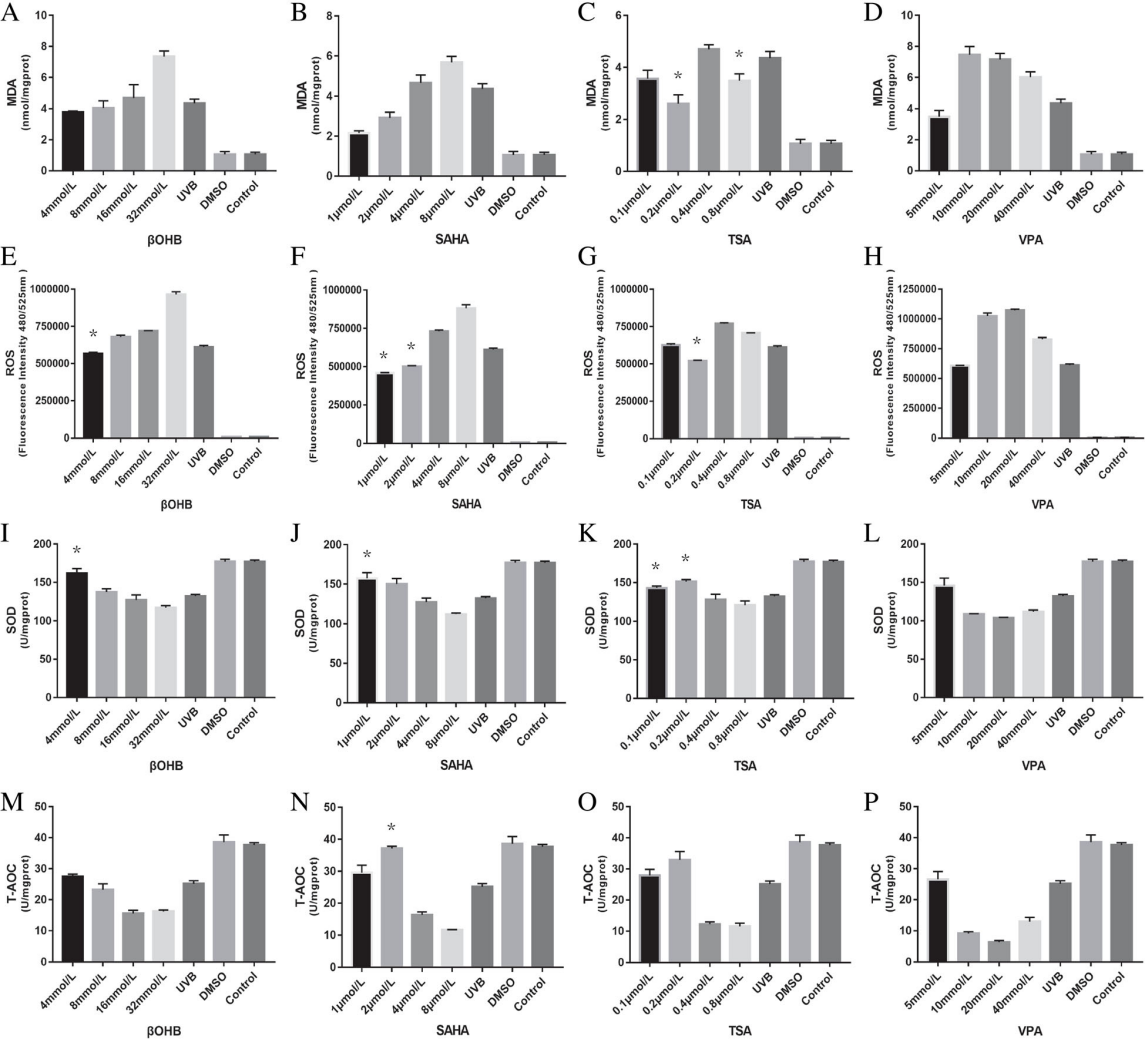
HDACi attenuated the oxidative stress in HLECs after UVB exposure. MDA:**a**-**d**, ROS: **e**-**h**, SOD**i**-**l**, T-AOC:**m**-**p**. MDA and ROS levels were relatively reduced while SOD and T-AOC levels were increased after HDACi administration. * *P* < 0.05

**Fig. 5:**
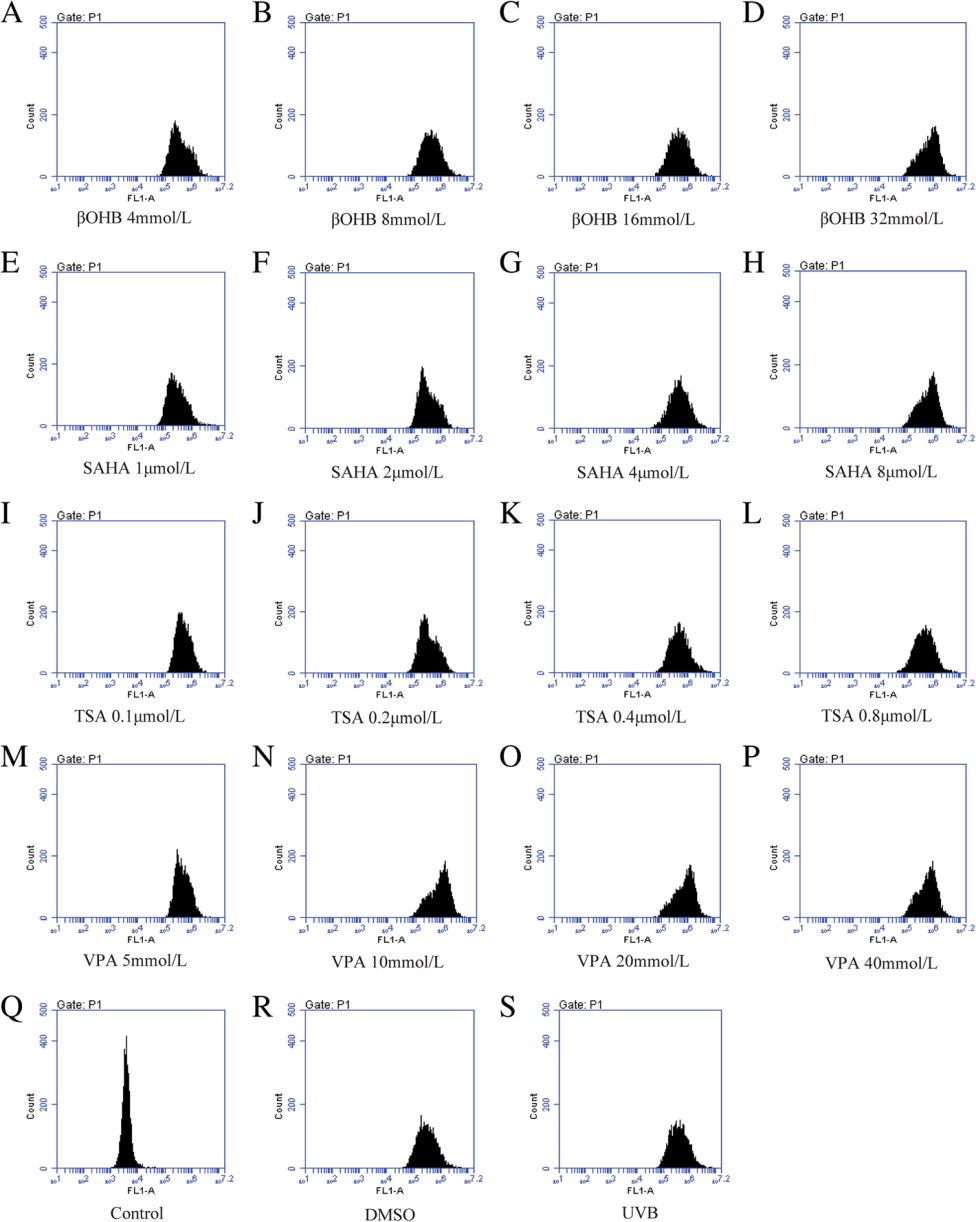
The ROS levels in HLECs treated with/without HDACi after UVB exposure βOHB:**a**-**d**, SAHA: **e**-**h**, TSA:**i**-**l**, VPA:**m**-**p**, Control group: Q, DMSO group: R, UVB group: S

As shown in Figs. 4 and 5, ROS and MDA levels were obviously enhanced in UVB-treated HLECs, while T-AOC and SOD levels were declined. However, after HDACi administration, MDA and ROS levels were relatively decreased while T-AOC and SOD levels were elevated. SOD levels were increased in UVB-treated HLECs following treatment with βOHB (4 mmol/L: *P* = 0.005), SAHA (1 μmol/L: *P* = 0.002) and TSA (0.1 mol/L: *P* = 0.013, 0.2 mol/L: *P* = 0.010). T-AOC levels were increased in UVB-treated HLECs following treatment with SAHA (2 μmol/L: *P* = 0.006). MDA levels were decreased in UVB-treated HLECs following treatment with TSA (0.2 mol/L: *P* = 0.003, 0.8 mol/L: *P* = 0.029). ROS levels were decreased in UVB-treated HLECs following treatment with βOHB (4 mmol/L: *P* = 0.008), SAHA (1 μmol/L: *P* = 0.001, 2 μmol/L: *P* = 0.001) and TSA (0.2 mol/L: *P* = 0.001).

### HDACis upregulate SOD1 expression levels and activity in HLECs

**Fig. 6:**
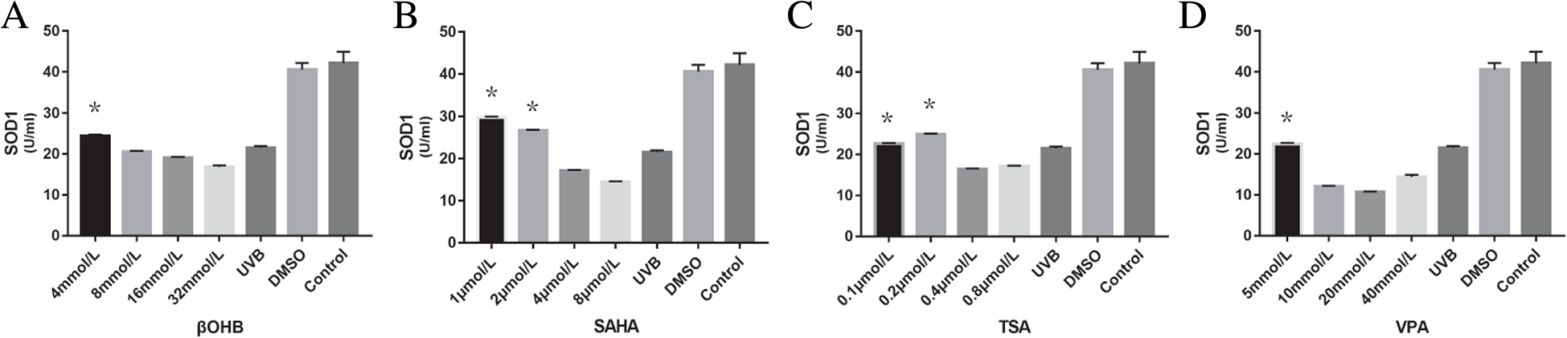
HDACi upregulated the expression level and activity of SOD1 in HLECs A: βOHB, B: SAHA, C: TSA, D: VPA. SOD1 levels significantly increased in the βOHB (4 mmol/L), SAHA (1 μmol/L, 2 μmol/L), TSA (0.1 mol/L, 0.2 mol/L) and VPA (5 mmol/L) groups after UVB exposure. * *P* < 0.05

**Fig. 7:**
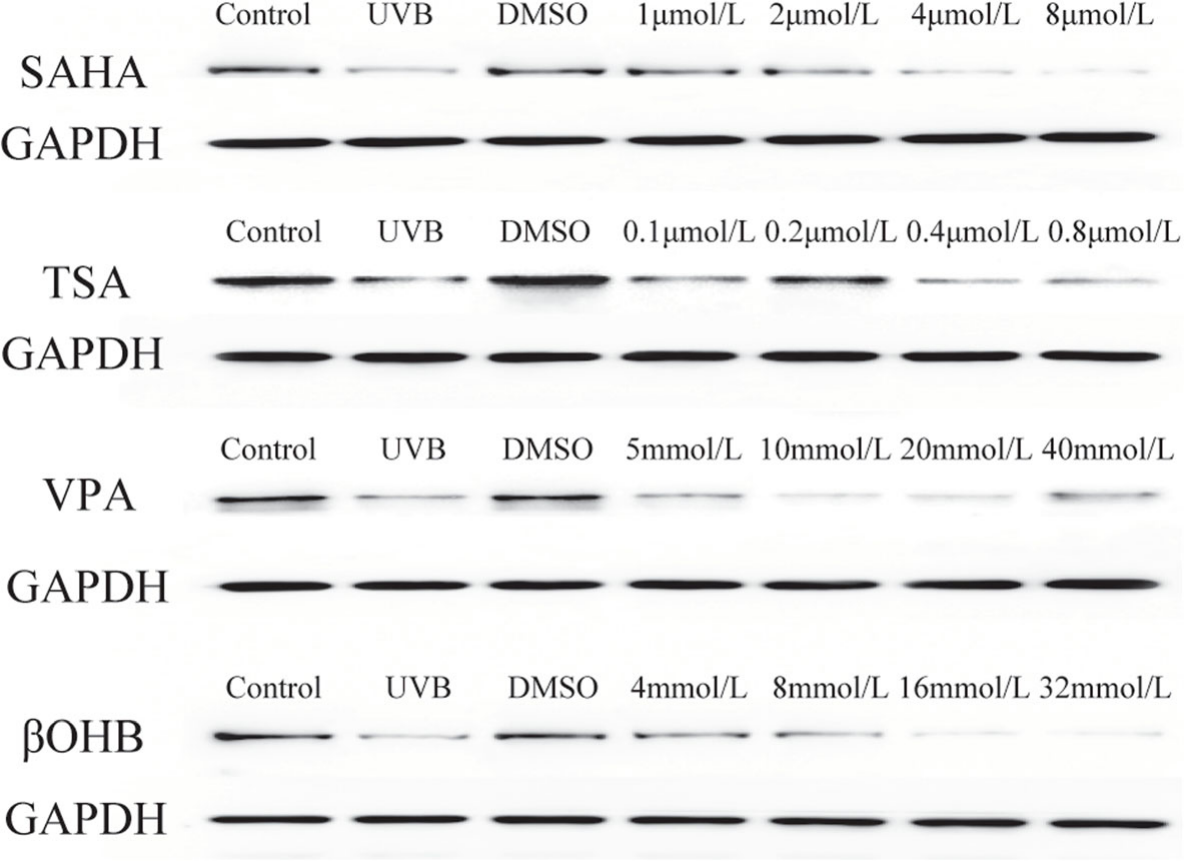
The effect of HDACi on on the protein expressions of SOD1

Western blotting results demonstrated that SOD1 expression levels were upregulated following HDACi administration (Figs. 6 and 7). SOD1 levels significantly increased in the βOHB (4 mmol/L: *P* = 0.005), SAHA (1 μmol/L: *P* = 0.001, 2 μmol/L: *P* = 0.004), TSA (0.1 mol/L: *P* = 0.013, 0.2 mol/L: *P* = 0.010) and VPA (5 mmol/L: *P* = 0.020) groups after UVB exposure.

### HDACis display mild anti-apoptotic effects in UVB-treated HLECs on MMP

**Fig. 8:**
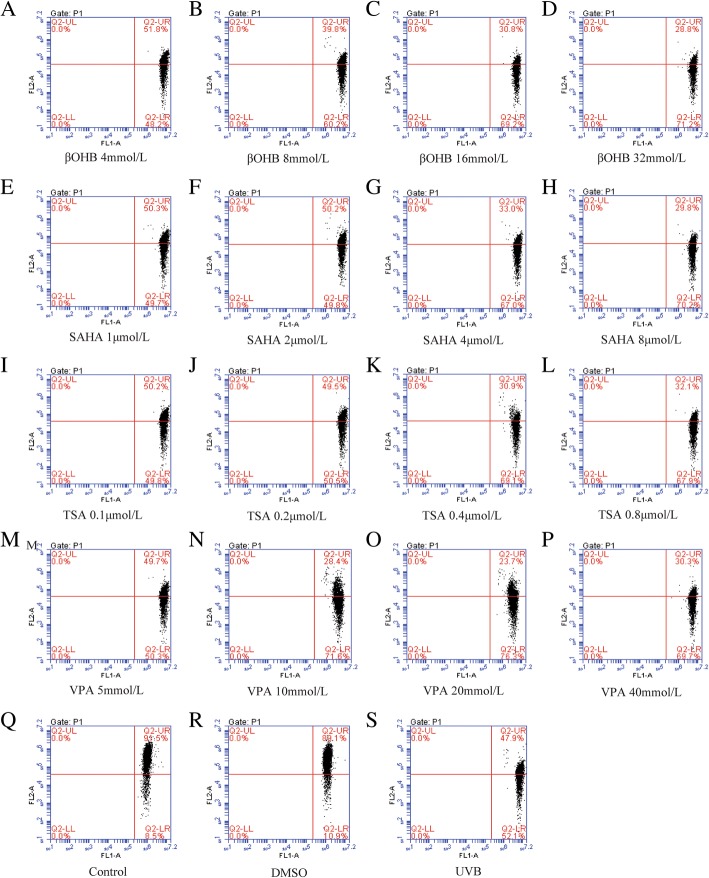
The effect of HDACi on MMP in HLECs after UVB exposure. βOHB:**a-d**, SAHA: **e-h**, TSA:**i-l**, VPA:**m-p**, Control group: **q**, DMSO group: **r**, UVB group: **s**. UVB exposure to cells facilitates the depolarization of the mitochondrial membrane. However, HDACis were not able to reduce the UVB-induced depolarization of the mitochondrial membrane

We also analyzed the effect of HDACis on MMP using JC-1. The control and DMSO-treated cells showed weak green fluorescence and strong red fluorescence, indicating high MMP. UVB exposure to cells accelerated the depolarization of the mitochondrial membrane, which manifested as stronger green fluorescence and. Weaker red fluorescence. However, we found that HDACis were not able to reduce the depolarization of the mitochondrial membrane induced by UVB exposure (Fig. 8). These results suggested that HDACis could not protect against oxidative stress-induced changes in MMP. Taken together, these results indicate that HDACis exert a potent anti-apoptotic effect on UVB-treated HLECs.

## Discussion

HLECs compose the cell layer of the eye that is first exposed to environmental and oxidative insult. Many pivotal factors are involved in the pathogenesis of cataracts, including oxidative stress. Oxidative stress plays an important role in the cross-linking and aggregation of lens proteins, oxidation, induction of lens epithelial cell apoptosis and aging [11]. Therefore, antioxidant treatments have been considered to treat cataracts [12]. HDACis boast a celebrated history: they were initially studied for their ability to increase gene expression [1] but in recent years have also been discovered to have potent immunomodulatory, anti-inflammatory, antioxidant, and anti-cancer functions [6–8]. These antioxidant properties suggest that HDACis may be potential therapies for age-related diseases including cataracts [17–19]. In our present study, we analysed the the potential effect of HDACis on cataracts. We used HLE-B3 cells in our experiments because these cells are considered the main cell type affected by UVB radiation and thus represent a potential target for intervention.

In our study, we evaluated the underlying effects of HDACis on UVB-injured HLECs. We selected four HDACis based on the potential to translate our findings into clinical studies. βOHB, which mainly produced by hepatocytes, serves as an alternative source of ATP in low-glucose conditions by carrying energy from the liver to peripheral tissues [20]. In recent studies, βOHB has been shown to act as an endogenous HDACi and to exert important cellular signaling effects [20–22]. βOHB treatment also elevates histone acetylation at the promoters of FOXO3A and MT2 involved in anti-oxidation [21]. SAHA (vorinostat, Zolinza®) is the lead compound in a new class of HDACis and is the first drug in this class to be clinically approved for the treatment of cancer. SAHA has been shown to affect multiple proteins associated with cell migration, proliferation and gene expression [23]. SAHA functions through multiple mechanisms, many of which involve its ability to serve as an NO donor under oxidizing conditions [24, 25]. TSA is a classical, noncompetitive and nonselective HDAC inhibitor and which derived from the Streptomyces species as a fermentation product [26]. A recent study found that TSA can suppress EMT and proliferation of the HLEC lines SRA01/04 and HLEB3, and can prevent the formation of posterior capsule opacification [27]. VPA, a class I HDAC inhibitor, is used clinically as an antiepileptic drug and for some painful neuropathies; it has a low toxicity profile and a complex pharmacological mechanism [28, 29]. Recent studies have reported that VPA exerts its antitumor effects in various cancers through HDAC inhibition and clinical studies of this drug are ongoing [30, 31]. The result of our study showed obvious variety in the effect of different HDACis on HLECs after UVB exposure, which might be explained by the mechanisms of actions are quite different among these four HDACis,

Accumulating evidence reveals that oxidative stress is a probable contributor to cataract formation [11, 12]. MDA levels reflect the extent of oxidative damage to cell membranes [32]. SOD is the first line of defense against ROS,and acts as an important factor in the antioxidant enzymatic defense system, which. T-AOC levels reflect the overall endogenous cellular antioxidant capability [33]. Hence, in the present study, SOD, ROS, MDA and T-AOC levels were evaluated to assess the oxidative stress in HLECs. We showed that, ROS and MDA levels were substantially elevated while T-AOC and SOD levels were decreased after UVB exposure. In contrast, HDACi treatment decreased MDA and ROS levels and simultaneously enhanced SOD and T-AOC levels. These results indicated that HDACis could decrease UVB-induced oxidative stress, which might lead to promising applications for cataract treatment.

Oxidative stress can also trigger the apoptosis of LECs. Previous studies have shown that increased FOXO3A expression elevates the cell’s ability to resist oxidative stress [34]. The proteins Bax and Bcl-2 are widely recognized as the most important regulators of apoptosis. The specific ratio of Bcl-2 to BAX gene expression is critical in determining cell fate [35]. Caspases are important indicators of apoptosis and caspase-3 is a major downstream effector caspase in the LEC apoptotic process [36]. Following UVB exposure, the extent of apoptosis as well as the transcription of BAX, caspase 3, FOXO3A and MT2 in HLECs were significantly increased, indicating that oxidative damage was stimulated. We also found that Bcl-2 and SOD1 transcription decreased in HLECs following UVB exposure, indicating that HLECs have antioxidant capabilities.

To explore the potential protective effects of HDACis on apoptosis in HLECs induced by UVB as a classical oxidative stress [37]. we used a range of HDACi concentrations as a dose-response experiment. The doses chosen for our experiments were based on published studies that reported the ranges required to inhibit HDACs [4–8]. The results indicated that low concentrations of HDACis exerted a certain extent of protective effects against oxidization and inhibitory effects against cell apoptosis. Our study noted that HDACi prevented UVB-induced elevation of BAX, caspase 3, Foxo3a and MT2, but not in a dose-dependent manner. This result indicates that HDACi is able to modulation of Bax/Bcl-2 expression in order to protect against UVB-induced apoptosis.

Several of the currently available HDACis have antioxidant properties, whereas associated with obvious toxicity [38]. This maily due to none class-specific inhibitor and complexity of cellular pathways involved [39]. Our study showed that high concentrations of HDACis manifested in high toxicity that outweighed that antioxidant effects. Identification of more selective HDACis with lower toxicity [38] will be important for antioxidant-based treatments with increased potency. As demonstrated in previous studies, significantly lower HDACi concentrations can be used in models of autoimmune diseases compared with those required to reduce tumors in mice [40]. These results indicate that lower concentrations may be sufficient to exert antioxidant effects.

The future development of HDACis should be focused on selective inhibitors [41]. It should also be expected that different HDACis will be most effective depending on the dominant cell type in a particular disease. Our findings as a preliminary study provided possible direction for future research. The exact mechanism of HDACis still need further investigation. More precise measurements were also needed to accurately evaluate the role of HDACis. Further studies should focus on determining the efficacy, safety, and pharmacokinetic properties of HDACis. Specific studies should also focus on identifying the metabolic pathways associated with HDACi antioxidant activities. Moreover, a comprehensive overview of HDACi-related adverse effects should be given.

## Conclusions

In summary, the current study provides direct evidence that few low concentrations of HDACis exert mild antioxidant effects on UVB-treated HLECs. These results suggest that HDACis may be a possible therapy for the prevention and treatment of cataracts.

## Abbreviations

ARCs: age-related cataracts
DMEM: Dulbecco’s modified Eagle’s medium
DMSO: dimethyl sulfoxide
ECL: enhanced chemiluminescence
ELISA: Enzyme-linked Immunosorbent Assay
EMT: epithelial mesenchymal transition
FBS: fetal bovine serum
FITC: fluoresceine isothiocyanate
HATs: histone acetyltransferases
HDACis: histone deacetylase inhibitors
HDACs: histone deacetylases
HLE-B3: human lens epithelium B3
HLECs: human lens epithelial cells
MDA: malondialdehyde
MMP: Mitochondrial Membrane Potential
MT2: Metallothionein
PAGE: polyacrylamide gel electrophoresis
PI: propidium Iodide
PMSF: phenylmethylsulfonyl fluoride
PVDF: polyvinylidine difluoride
ROS: reactive oxygen species
SAHA: suberoylanilide hydroxamic acid
SOD: superoxide dismutase
T-AOC: total antioxidant capacity
TSA: trichostatin A
UVB: ultraviolet-B
VPA: valproic acid
βOHB: β-hydroxybutyrate
